# The cost-effectiveness analysis of the New Rural Cooperative Medical Scheme in China

**DOI:** 10.1371/journal.pone.0208297

**Published:** 2018-12-10

**Authors:** Jinjing Wu, Shelby Deaton, Boshen Jiao, Zohn Rosen, Peter A. Muennig

**Affiliations:** 1 Asian Demographic Research Institute, Shanghai University, Shanghai, People’s Republic of China; 2 Global Research Analytics of Population Health, Columbia University, New York, New York, United States of America; 3 The Comparative Health Outcomes, Policy, and Economics (CHOICE) Institute, University of Washington, Seattle, Washington, United States of America; 4 Department of Health Policy and Management, Columbia University, New York, New York, United States of America; NPMS-HHC CIC / LSH&TM, UNITED KINGDOM

## Abstract

**Objective:**

The New Rural Cooperative Medical Scheme (NCMS) is a universal healthcare coverage plan now covering over 98% of rural residents in China, first implemented in 2003. Rising costs in the face of modest gains in health and financial protections have raised questions about the cost-effectiveness of the NCMS.

**Methods:**

Using the most recent estimates of the NCMS’s health and economic consequences from a comprehensive review of the literature, we conducted a cost-effectiveness analysis using a Markov model for a hypothetical cohort between ages 20 and 100. We then did one-way sensitivity analyses and a probabilistic sensitivity analysis using Monte Carlo simulations to explore whether the incremental cost-effectiveness ratio (ICER) falls below 37,059 international dollars [Int$], the willingness-to-pay (WTP) threshold of three times per capita GDP of China in 2013.

**Findings:**

The ICER of the NCMS over the lifetime of an average 20-year-old rural resident in China was about Int$71,480 per quality-adjusted life year (QALY) gained (95% confidence interval: cost-saving, Int$845,659/QALY). There was less than a 33% chance that the system was cost-saving or met the WTP threshold. However, the NCMS did fall under the threshold when changes in the program costs, the risk of mortality and hypertension, and the likelihood of labor force participation were tested in one-way sensitivity analyses.

**Conclusion:**

The NCMS appears to be economically inefficient in its current form. Further cost-effectiveness analyses are warranted in designing insurance benefit packages to ensure that the NCMS fund goes toward health care that has a good value in improving survival and quality of life.

## Introduction

By pooling financial risk, health insurance aims to alleviate economic barriers to accessing health care, thereby improving health and longevity [1,2,3]. The Cooperative Medical Scheme (CMS) was the primary system for providing basic health care to rural residents during the 1950s and 1970s [4]. However, the market economy reform led to the collapse of both collective farming and the CMS, leaving the majority of rural residents uninsured in the 1990s [5,6,7].

In 2003, China initiated the New Rural Cooperative Medical Scheme (NCMS) [6]. By 2013, the NCMS had enrolled 98.9% of all rural residents [8]. The NCMS is financed by a combination of individual users and government funds [9,10]. Within this system each county is responsible for designing their own benefit packages [10]. Therefore, the coverage and generosity of the scheme vary by counties [10,11,12]. For example, all counties reimburse inpatient care, but not all reimburse outpatient care since the initiation of NCMS [12]. More details about the scheme’s regional heterogeneity could be found in [11,13].

The NCMS was intended to promote health services use, alleviate financial burdens on families, and improve rural residents’ overall survival and quality of life. However, there is considerable uncertainty as to whether it succeeded in doing so. Several studies suggested that the scheme increased the probability of seeking health care [11,12,14], while others demonstrated that it was not clearly related to the healthcare use [7,15]. Its financial protections have been found to be modest at best [9,16,17,18]. The NCMS has also resulted in some medical inflation, leading to a 61% increase in out-of-pocket spending since its implementation and potentially negating the protective effect of risk pooling [14].

A series of quasi-experimental studies assessing the health consequences of the scheme also produced mixed results [7,19,20,21]. Using data from twenty-two provinces, the scheme was estimated to be associated with a 9.4% reduction in three-year mortality among seniors in the eastern region [20]. However, for the sample as a whole, changes in mortality rates were more modest (3.7%) [20]. The most recent study, using nationally representative data from the Disease Surveillance Point system of the Chinese Center for Disease Control and Prevention, found no statistically significant correlation between the implementation of NCMS and reductions in mortality [21]. However, the large standard errors of the estimated NCMS coefficients indicate that it is still possible the scheme had a positive effect on mortality. Also, the NCMS may affect the quality of life by decreasing the probability of having hypertension, although these effects are modest [20]. Moreover, the scheme may produce economic benefits by increasing hours worked in agriculture and the probability of working in the off-farm sector [22].

Given the considerable uncertainty surrounding the health and economic consequences of NCMS, our goal was to ascertain whether, under a range of assumptions, the health gains associated with NCMS have been worth the investment. Answering this question is of critical importance from a policy standpoint because the NCMS is the primary insurance scheme for a considerable proportion of the earth’s inhabitants. Our model was designed to test its performance concerning mortality reduction, hypertension prevention, and the increase in total health expenditures as well as labor productivity, as well as to discover which aspects would produce the most considerable improvement in the value of NCMS.

## Methods

### Overview and definitions

We built a Markov model to capture the lifetime health and economic effects of NCMS for a hypothetical cohort of 20-year-old rural residents in China. The simulated participants were then followed until their death, or until they reached 100. We evaluated the cost-effectiveness of the NCMS from a societal perspective and considered its effects on the risk of mortality and hypertension, total health expenditure per rural resident, while including the indirect effects associated with increased labor force participation.

We assessed the health effects of the NCMS using quality-adjusted life years (QALYs), which combines both quantity of life and health-related quality of life (HRQL) [23]. An HRQL score is a multi-dimensional indicator used to quantify the effect of diseases on quality of life, ranging from 0 (equal to death) to 1 (equal to full health) [23]. A QALY is one year of life in perfect health [23].

According to the WHO guide cost-effectiveness analysis [24], and recommendations from the Panel on Cost-Effectiveness in Health and Medicine [25], an annual discounting rate of 3% was used to discount future economic costs, benefits, and QALYs. We converted costs and economic benefits into 2013 international dollars [Int$] using the purchasing power parity (PPP) exchange rate (Int$1.00≈3.55 CNY) obtained from the World Bank [26].

The incremental cost per QALY gained is computed as the incremental costs (including the increase in the total health expenditure, and savings from higher labor productivity) divided by the incremental QALYs. This ratio is known as the incremental cost-effectiveness ratio (ICER). According to the World Health Organization (WHO), interventions that have an incremental cost-effectiveness ratio of fewer than three times gross domestic product (GDP) per person are regarded as cost-effective [27]. The per capita GDP of China in 2013 after the PPP adjustment was Int$12,353 [28]. Therefore we used Int$37,059 per QALY as the willingness-to-pay (WTP) threshold to determine whether the NCMS was cost-effective.

### The NCMS’s effectiveness

#### Mortality reduction

To measure the NCMS’s potential effect on survival, we created a hypothetical cohort which was then exposed to our calculated hazard of mortality in sequential annual cycles. The background age-specific mortality rates were obtained from the life table of China in 2013 (See Table A in S1 File) [29]. For the change in mortality rates attributable to the NCMS, we used the figures estimated by Zhou and his colleagues [21]. Their approach, using data from the Disease Surveillance Point system, arguably represents the most sound data source exploring mortality associated with the NCMS currently available. While this study found that the implementation of NCMS did not statistically reduce mortality rates, the very broad confidence intervals (particularly in the older age groups) suggested the possibility that the NCMS had effects on rural residents’ survival. For example, males aged 60 years or older saw a 0.36 (95% confidence interval [CI]: -3.01, 3.73) increase in age-standardized mortality rates per 1000 population (see Table B in S1 File for all other age group coefficients) [21].

#### Hypertension prevention

The NCMS may increase QALYs by lowering the risk of having hypertension [20,30] as the HRQL score in hypertensive people is meaningfully lower than that of normotensive people [31].

To assess the number of incremental QALYs gained due to the NCMS, we added two health states, a hypertensive state and a normotensive state into the Markov model. The hypothetical cohort was then exposed to the probability of hypertension. We reported the age- and sex-specific likelihood of hypertension in Table C in S1 File [32,33].

### The NCMS’s costs

The increase in out-of-pocket medical payments due to the NCMS was about 61% of the baseline mean in the rural counties in which it was enacted [14]. We assumed that the health spending from governments and social health insurance funds also increased proportionally by 61%. Further, we assumed that the growth in total health expenditures includes increases in medical utilization, supplier-induced demand, and deadweight loss associated with taxing and administering the plan.

According to the China Health Statistical Yearbook, the total health expenditure per rural resident in 2013 was Int$359 [8], which was inclusive of both public and private contributions. We also estimated the total health expenditure per rural resident by provincial area (see Table D in S1 File). We entered the estimates in Beijing (Int$977) and Guizhou (Int$243) [8] into our analysis to evaluate the effects of very low and high total health expenditure per capita.

Given that the NCMS covered 98.9% of rural residents in 2013 [8], we estimated the increase in total health expenditure per rural resident due to the NCMS through the formula below:

$$Additional\;health\;expenditures\;=\;{HE}_{1}-{HE}_{0}\;=\;Int\$359-\frac{Int\$359}{1.61}\approx Int\$136$$

where HE_1_ represents the total health expenditure per rural resident after implementing the NCMS, and HE_0_ represents that figure before the NCMS. Therefore, we assumed that the total health expenditures per rural resident would be increased by approximately Int$136 (Int$370 in Beijing, and Int$92 in Guizhou) every year due to the NCMS.

### The NCMS’s economic benefits

#### Savings from hypertension prevention

The NCMS may reduce the probability of having measured hypertension [20,30], which could save medical costs associated with hypertension and related comorbidities. The average annual direct medical expense among hypertensive patients in rural China was estimated to be Int$392 (95% CI: Int$344, Int$441) in 2013 [34]. We did not include indirect costs of hypertension such as transportation and caregiver costs.

#### Savings from labor productivity increase

The implementation of NCMS may also raise the labor productivity by increasing hours worked in agriculture and the probability of working in the off-farm sector [22]. We reported the details about the calculation of economic benefits associated with labor productivity increases in Table E in S1 File.

### Statistical analysis

We built a Markov model to estimate the incremental cost and effectiveness of providing the NCMS to a hypothetical cohort of 20-year-old rural residents in China relative to the absence of NCMS. The simulated participants were followed until their death or until they reached 100 years of age. The model contained two arms—NCMS versus no NCMS—and it included three health states: no hypertension, hypertension, and death. The hypothetical cohort was exposed to the age-specific probabilities of mortality and hypertension during each one-year life cycle. If the simulated participants died, they exited the model. We assumed that those who got hypertension would stay in the hypertensive state, and thus constantly lose HRQL and incur hypertension-related medical costs for the rest of their life.

To evaluate the uncertainty of the model, we did a series of one-way sensitivity analyses, and conducted a probabilistic sensitivity analysis using Monte Carlo simulations (10,000 simulations). We examined how varying the NCMS’s effect on the risk of mortality and hypertension, hours worked in agriculture, and the probability of working in the off-farm sector impacted model results. We also varied the total health expenditure per rural resident, the growth rate in the total health expenditure due to the NCMS, the HRQL score of the hypertensive and normotensive, the hypertension-related medical cost, the annual wage of rural resident, and hourly income for agricultural workers to measure the impact of these inputs on overall model results.

The Monte Carlo simulation allowed us to compute the probability that the ICER is below the WTP threshold. The model parameter inputs are presented in Table 1, including the baseline values, and assigned low and high values of the variables which were used to create triangular distributions for the Monte Carlo simulation. Model assumptions are listed in Table 2. We conducted all analyses using TreeAge Pro 2017 (TreeAge Software, Williamtown MA).

**Table 1:** Model inputs used in the analysis comparing NCMS and no NCMS * (NCMS = New Rural Cooperative Medical Scheme).

Parameters	Base	Low	High	Source
**Probabilities, rates, or risk ratio**				
Marginal Effects of NCSM on age-standardized mortality rate per 1,000 population †				
Male, aged 20–44 years	-0.10	-0.28	0.08	Zhou et al., 2017
Male, aged 45–59 years	-0.14	-0.57	0.29	
Male, aged 60 and above	0.36	-3.01	3.73	
Female, aged 20–44 years	-0.05	-0.12	0.03	
Female, aged 45–59 years	-0.05	-0.32	0.23	
Female, aged 60 and above	1.05	-1.72	3.82	
Risk ratio of being hypertensive for the NCMS group	0.98	0.95	1.00	Cheng et al., 2015
Probability of off-farm labor participation	0.17			Shen et al., 2017
Increase in probability of off-farm labor participation				
Aged 30–49 years	0.13	0.07	0.20	Shen et al., 2017
Aged 50 years or more	0.07	0.03	0.11	
Health expense growth rate ‡	0.61	0.50	0.70	Wagstaff et al., 2009
Annual discount rate for economic costs, benefits, and quality-adjusted life-years	0.03	0.00	0.05	Weinstein et al., 1996
Increase in probability of off-farm labor participation				
Aged 30–49 years	0.13	0.07	0.20	Shen et al., 2017
**Utilities (Health-related quality of life score)**				
Normotensives	0.98	0.98	0.98	Zhang et al., 2017
Hypertensives	0.92	0.90	0.94	
**Costs or benefits, 2013 International dollars** §				
Total health expenditure per rural resident	359	243	977	CMOH, 2014
Hypertension-related medical costs per year	392	344	441	Liang et al., 2011
Annual wage per rural resident ||	1029	350	4198	NBS, 2015
Income per hour in agriculture	1.44	0.47	2.59	NBS, 2015 & Shen et al., 2017
Increase in hours of working in agriculture per year				
Aged 30–49 years	1.10	0.84	1.44	Shen et al., 2017
Aged 50 years and above	1.28	1.07	1.53	

* The baseline values are the most likely. We used the high and low values in one-way sensitivity analyses and a Monte Carlo simulation.

^†^ Estimated by Zhou and their colleagues [21].

^‡^ The baseline value was based on a quasi-experimental study done by Wagstaff and his colleagues [14]. The high and low values were bounded by the team consensus as to the most plausible values.

^§^ 1 international dollar = 3.55 CNY.

^||^ We obtained the data of annual wage per rural resident from the 2015 China Rural Statistical Yearbook [35].

**Table 2:** Assumptions used in the Markov model evaluating the implementation of NCMS versus no NCMS in China.

1. The health-related quality of life (HRQL) scores among hypertensive and normotensive individuals were based on an observational study in Shandong Province, China [31]. We assumed that these HRQL scores were generalizable to all rural residents in China.
2. The risk ratio of having measured hypertension in the NCMS group was based on a natural experimental study, with data from a nationally representative sample of people aged 60 years or above [20]. We assumed that the risk ratio was generalized to adults at least 20 years old. Also, we assumed that people who have no measured hypertension are at normotensive state.
3. We assumed that once simulated participants get hypertension, they would remain hypertensive, and would be subject to a decrease in the HRQL score and incur hypertension-related medical expenses for the rest of their life.
4. We assumed that having hypertension did not increase the risk of mortality in the hypothetical cohort.

## Results

The results for the base case were summarized in Table 3. From the societal perspective, the implementation of NCMS in rural China increases the discounted costs by Int$825 per rural resident over the 80-year interval from age 20 to 100. The average 20-year-old person would gain 0.01 QALY over this interval, resulting in an ICER of 71,480/QALY at a discounted rate of 3%.

**Table 3:** The base-case cost-effectiveness analysis of the NCMS versus no NCMS (NCMS = New Rural Cooperative Medical Scheme).

	Cost, Int$ *	Incremental cost, Int$ *	Effectiveness, QALYs †	Incremental effectiveness, QALYs †	ICER, Int$ */QALY †
No NCMS	1,010		14.21		
NCMS	1,836	825	14.22	0.01	71,480

* Int$ = international dollars. 1 International dollar = 3.55 CNY.

^†^ QALY = quality-adjusted life year.

In the probabilistic sensitivity analysis with Monte Carlo simulations (See Table F in S1 File), the gains for the average participant were approximately 0.01 (95% CI: -0.08, 0.10) QALYs, and the additional cost attributable to the NCMS was Int$1,013 per rural resident (95% CI: -2,773, 5,363), indicating that the 95% plausible interval of the ICER ranged from cost-saving to Int$845,658 per QALY gained.

The 95% CI of the effectiveness and the ICER includes negative values which suggest the possibility that the NCMS resulted in a loss in QALYs. This is because Zhou et al. (2017) found that the estimated NCMS coefficients on mortality among some age groups were positive, but with extremely broad confidence intervals [21]. We do not believe that it is plausible that the NCMS increases mortality. However, truncating values at zero would artificially inflate the ICER estimates that we generate using our Monte Carlo simulation, so we left the intervals intact.

**Fig 1:**
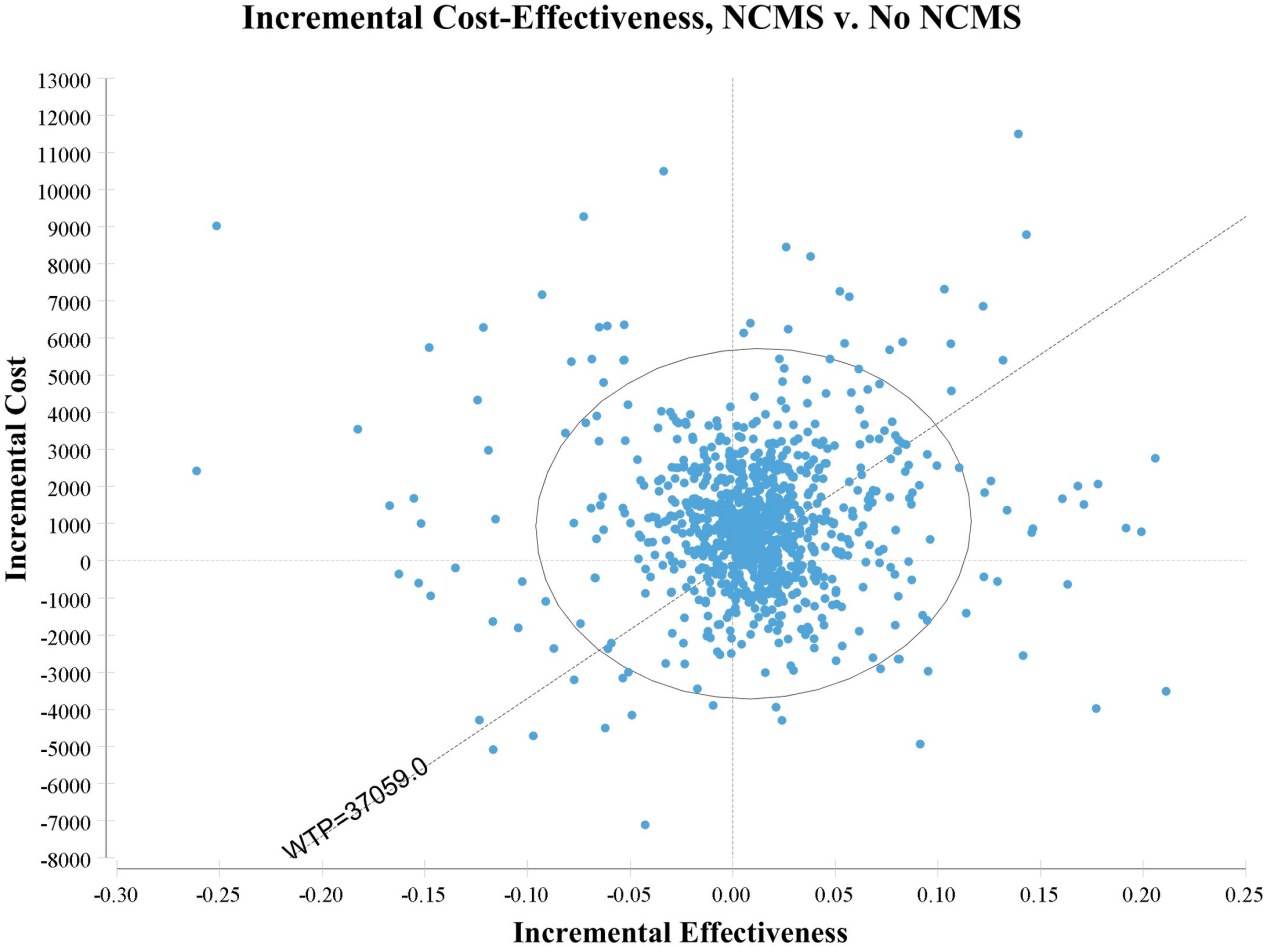
The scatterplot of incremental cost-effectiveness ratio comparing the NCMS to no NCMS (NCMS = New Rural Cooperative Medical System). The willingness-to-pay (WTP) threshold is three times per capita GDP (Int$37,059 after the purchasing power parity adjustment. 1 international dollar = 3.55 CNY).

Fig 1 shows the probability that the NCMS was cost-effective relative to the WTP threshold of three times per-person GDP as of 2013 in China (Int$37,059). The NCMS had a 17.5% chance of being dominant and a 15.3% chance of being cost-effective. However, it also had a 34.8% chance of being not cost-effective and a 32% chance of resulting in a loss in QALYs (See Table G in S1 File).

Based on the low and high values of the parameters shown in Table 1, the results of one-way sensitivity analyses for the most influential variables were reported in Table 4. We presented the results for the other variables in Table H in S1 File. The model results were most sensitive to the total health expenditure per rural resident, where the ICER ranged from cost saving to Int$313,855 per QALY gained. The model results are also sensitive to the growth rate in the total health expenditure per rural resident. When the growth rate was 50%, the ICER decreased to Int$43,141 per QALY, although that figure is still beyond the WTP threshold. But if the growth rate could drop to 48%, the ICER (Int$36,462/QALY) would fall below the threshold.

**Table 4:** One-way sensitivity analyses of the NCMS versus no NCMS (NCMS = New Rural Cooperative Medical Scheme).

Parameters	Incremental cost, Int$ *		Incremental effectiveness, QALYs †		Effect on ICER, Int$ */QALY †	
	Low	High	Low	High	Low	High
Marginal Effects of NCSM on Mortality, Males 20–44	909	910	0.04	-0.01	25,300	Dominated
Marginal Effects of NCSM on Mortality, Males 45–59	910	908	0.03	-0.001	36,207	Dominated
Marginal Effects of NCSM on Mortality, Males 60+	915	903	0.05	-0.03	17,192	Dominated
Marginal Effects of NCSM on Mortality, Females 20–44	909	909	0.02	0.002	41,534	382,543
Marginal Effects of NCSM on Mortality, Females 45–59	910	908	0.02	0.004	44,035	251,646
Marginal Effects of NCSM on Mortality, Females 60+	915	904	0.05	-0.02	18,121	Dominated
Risk ratio of having hypertension for the NCMS group	863	950	0.02	0.01	45,728	157,694
Total health expenditure per rural resident	-911	3805	0.01	0.01	Cost-saving	313,855
Growth rate in the total health expenditure per rural resident	523	1,228	0.01	0.01	43,141	101,285
Increase in the probability of off-farm labor participation						
Aged 30–49 years	1749	43	0.01	0.01	144,289	3,532
Aged 50–59 years	1276	524	0.01	0.01	105,247	43,188
Annual wage per rural resident	2865	-2122	0.01	0.01	236,304	Cost-saving
Discount Rate	2540	478	-0.01	0.01	Dominated	68,012

* Int$ = international dollars. 1 International dollar = 3.55 CNY.

^†^ QALY = quality-adjusted life year.

The results also highlight the impact of changes in mortality rates on the ICER. For instance, if the mortality rate among females aged 60 years or above could be reduced by 1.72 per 1,000 people, the scheme would be deemed cost-effective. However, if we assumed that this group’s mortality rate was increased by 3.82 per 1,000 people, the NCMS would not only increase costs, but also reduce QALYs. But as stated above, we do not believe that it is plausible that the NCMS could have increased mortality.

The results also depended on the risk ratio of being hypertensive for the NCMS group. If we assumed that there was no decreased risk of hypertension due to the NCMS, the ICER would rise to Int$157,694 per QALY. But if we assumed that the risk ratio was 0.95, the ICER would decrease to Int$45,728 per QALY. And when the risk ratio decreased to 0.93, the ICER would be Int$34,202 per QALY, which is below the WTP threshold.

The model results are also sensitive to the change in the labor productivity. When the annual wage per rural resident was increased to Int$4,198, the NCMS was cost-saving. Furthermore, the scheme would be cost-effective if the NCMS could increase the probability of off-farm work participation among rural residents aged between 30–49 years by approximately 20%.

## Discussion

From the societal perspective, the NCMS plausibly produced up to one additional QALY at the cost of Int$71,480 (95% CI: cost-saving, Int$845,659/QALY). If Chinese policymakers adhere to three times per capita GDP as a WTP threshold (Int$37,059/QAL), then the system appears to come at a poor value. Based on the best data available to date, there was less than a 33% chance that the system was dominant or met the WTP threshold. However, if the NCMS could reduce mortality among senior adults, then the plan could be valued within the range of Int$37,059/QALY gained. Additionally, if the NCMS’s efficacy of hypertension prevention could be enhanced, the scheme would become cost-effective as well. Moreover, if the growth rate in health expenditure due to the NCMS could drop to 48% or below, the NCMS would also become cost-effective.

To control the demand-side moral hazard and sustain the limited funding of NCMS, some local governments had set up the program with low reimbursement rates [36], low ceilings for maximum payouts [37], and narrow benefit packages with some procedures, medicines, and even outpatient care not being covered [38]. However, our findings from sensitivity analyses suggest that, while necessary, demand-side cost control measures might not be enough to bring NCMS to a price point that would be considered a good value. It appears that redirecting financial resources to produce more considerable gains in life expectancy and quality of life would improve the efficiency of NCMS.

The scheme was introduced to serve as a catastrophic health insurance plan, which was skewed toward inpatient expense reimbursements [37,39]. The limited effect of the scheme on mortality and morbidity might be attributable to this arrangement because it may lead people to skimp on preventive care or medically necessary diagnostic tests, resulting in severe illness and costly complications in the future. More counties, however, have offered coverage for outpatient care since 2008 [40]. Further research is needed to evaluate the scheme’s impact on mortality and quality of life as the scheme has changed over recent years.

The launch of the NCMS took place during a dramatic transformation in the social and economic conditions in China. This, coupled with an aging population, had led to an epidemic of non-communicable diseases [41,42], making primary care essential to prevent and detect chronic diseases, and to maintain quality of life among the chronically ill [42,43,44]. We found that if the NCMS’s efficacy of hypertension prevention could be enhanced to the point where the risk ratio of becoming hypertension is 0.93 or below, the scheme would meet the WTP threshold. This indicates that additional investments in primary care might be one solution, and some additional systemic investments might produce more value for the program overall. Shifting the funding emphasis from tertiary care to primary care may be one way of reducing costs while increasing effectiveness. Further studies on the impacts of such a shift on catastrophic medical expenses are needed.

Health interventions programs like the NCMS can do more than provide clinical services [45]. This additional service could include interventions for expectant parents, or cross-sectoral linkages to investments that might traditionally fall outside of the health system (e.g., farm safety education) that can be nevertheless integrated into the NCMS to improve its reach and efficiency.

Another strategy might be to make additional investments in the social determinants of health [46]. Given the high rates of smoking in rural China [47], improvements in health might be realized by smoking taxation [48]. Also, there is considerable room for improvement in rural education systems and social insurance schemes. Investments in these areas could produce long-term improvements in population health, and therefore reductions in health system costs [49].

Another element that should be addressed are the high co-payments associated with the scheme [36,37,50]. Based on the findings of several quasi-experimental studies, the NCMS failed to reduce participants’ out-of-pocket expenses per outpatient visit or hospitalization admission [14], and reimbursement policies for chronic diseases did not provide meaningful protections against catastrophic payments [51]. The failure of NCMS to provide financial protection against catastrophic illness ultimately restricts rural residents’ access to healthcare. Low premiums and high copayments mean that wealthy participants were more likely to utilize health services compared with poor ones, even though the latter generally have poorer health [43,52,53], which may limit the efficiency of the NCMS.

At the time when the NCMS was established, there has been an explicit tradeoff between the scope of coverage and generosity of benefits. As the economy of China improves, the financing level of the NCMS is expected to increase. To ensure that the NCMS fund goes toward health care that has a good value in improving survival and quality of life, the Chinese government should consider building an assessment into the rollout of expanded coverage, ideally exploiting the substantial geographic variation in plan implementation to experiment with different cost structures and investments.

Also, more centralized regulation and monopsony power may improve the efficiency of NCMS. Some researchers suggested that the county-level financing should be increased to improve NCMS’s risk sharing capacity to shield against financial risks of diseases [10,16,17] and that the horizontal consolidation of the rural and urban schemes is necessary [54]. Further research is required to investigate the appropriate risk pooling level for the NCMS [7].

And last but not the least, the reimbursement system may erode the efficiency of NCMS. Within the NCMS most health providers are paid on a fee-for-service basis [16]. At present, public facilities are under-funded, and these facilities sometimes attempt to recoup costs via inflating prices or providing unnecessary care including tests, treatments, and medications [38]. Our findings indicated that if the growth rate in health expenditure due to the NCMS could drop to 48% of the total health expenditure per rural resident, the system would become cost-effective. Some counties have reformed the payment structure by giving a fixed annual budget to providers combined with quota payments for specific diseases [55,56]. Such programs could be expanded.

This study essentially serves as a “thought experiment”, asking whether it is plausible that the current system produces reasonable value. Nevertheless, some limitations should be kept in mind. First, we obtained the estimate of the effect of NCMS as an aggregate of all of the data from different regions with markedly different implementations of NCMS as well as various social and economic demographics [21]. While these estimates do follow the trend of other literature, there are some regions and some age groups which have shown more promising outcomes, like the elderly in the Eastern region as reported in [20]. Second, we based our analyses on estimates from the existing literature. As the NCMS has developed and more counties have covered outpatient care since 2008 [40], further quasi-experimental studies are needed to derive any solid conclusions about the NCMS’s effect on rural residents’ mortality, quality of life, and other critical model inputs.

## Conclusions

China has made remarkable strides in making health insurance accessible to its rural residents. However, having access to health insurance is not the same as having access to affordable and high quality healthcare. China’s present expansion of the NCMS should consider the use of cost-effectiveness analysis in the design of benefit package, trade-offs between financing inpatient and outpatient services, incorporating non-clinical health interventions, improvements in financing structures, enlarging risk pools, and a move away from a fee-for-service system (or a more regulated fee-for-service system). These are just a few suggestions that may improve the efficiency of the system. China made the bold step of creating a good deal of local variation in the design and implementation of NCMS. Now is the time to exploit this variation to experimentally study which of these changes might work, and which might not.

## Supporting information

S1 File Supporting information on the cost-effectiveness analysis of the New Rural Cooperative Medical Scheme in China. (DOCX)
