# Supplementary material for: The cost-effectiveness analysis of the New Rural Cooperative Medical Scheme in China
Source: PLoS One. 2018 Dec 10;13(12):e0208297. doi: 10.1371/journal.pone.0208297 (PMC6287900; doi:10.1371/journal.pone.0208297)
Supplement: S1 File — (DOCX) [file pone.0208297.s001.docx]

**Supporting information on the cost-effectiveness analysis of the New Rural Cooperative Medical Scheme in China**

Table of Contents

[**1. 2013 World Health Organization Chinese life table** 2](#_Toc515773205)

[**2. The NCMS’s impact on mortality** 4](#_Toc515773206)

[**3. The incidence of being hypertensive** 5](#_Toc515773207)

[**4. The estimation of total health expenditure per rural resident by provincial region** 6](#_Toc515773208)

[**5. The NCMS’s effect on labor force participation** 10](#_Toc515773209)

[**6. Supporting information on the cost-effectiveness analysis** 12](#_Toc515773210)

[6.1 Results of probabilistic sensitivity analysis 12](#_Toc515773211)

[6.2 Incremental cost-effectiveness scatterplot report 13](#_Toc515773212)

[6.3 One-way sensitivity analyses 14](#_Toc515773213)

[**7. Reference** 15](#_Toc515773214)

# **1. 2013 World Health Organization Chinese life table**

The World Health Organization (WHO) provided gender-specific and abridged period life table of China in 2013 [1](WHO, 2015). However, death intervals of the life table did not match the cycle length of the Markov model in our study. We use the estimated mortality between ages x and x+n by using sex-specific mortality from the abridged Chinese life table and age-specific sex structure from the China Population and Employment Statistics Yearbook [2]. For the New Rural Cooperative Medical Scheme (NCMS) arm of the model, the age adjusted death rate coefficients estimated from Zhou, et. al. [3] are added to age-specific mortality rates before discounting. We excluded younger ages as Zhou’s estimates of the NCMS’s effects began at age 20. Table A shows sex and age-specific mortality rates among Chinese in 2013.

**Table A. Sex and age-specific mortality rates among Chinese in 2013**

|  | **nMx - age-specific mortality between ages x and x+n** * | | **Age specific sex**  **structure** † | |
| --- | --- | --- | --- | --- |
| **Age** | **Female** | **Male** | **Female (%)** | **Male (%)** |
| 20-24 | 0.000533 | 0.000612 | 0.488578955 | 0.511421045 |
| 25-29 | 0.000555 | 0.000638 | 0.496786604 | 0.503213396 |
| 30-34 | 0.000641 | 0.000717 | 0.49107506 | 0.50892494 |
| 35-39 | 0.000862 | 0.000994 | 0.488804546 | 0.511195454 |
| 40-44 | 0.001392 | 0.001734 | 0.489338157 | 0.510661843 |
| 45-49 | 0.002147 | 0.002929 | 0.490862039 | 0.509137961 |
| 50-54 | 0.003492 | 0.004942 | 0.490374055 | 0.509625945 |
| 55-59 | 0.005914 | 0.008765 | 0.493914822 | 0.506085178 |
| 60-64 | 0.010523 | 0.015251 | 0.496130045 | 0.503869955 |
| 65-69 | 0.018523 | 0.026098 | 0.501636552 | 0.498363448 |
| 70-74 | 0.032223 | 0.043912 | 0.504304694 | 0.495695306 |
| 75-79 | 0.055159 | 0.072022 | 0.523551833 | 0.476448167 |
| 80-84 | 0.098976 | 0.123394 | 0.553140693 | 0.446859307 |
| 85-89 | 0.163689 | 0.194074 | 0.611087866 | 0.388912134 |
| 90-94 | 0.28593 | 0.327174 | 0.663655316 | 0.336344684 |
| 95-99 | 0.430812 | 0.476082 | 0.766784452 | 0.233215548 |
| 100+ | 0.590169 | 0.630123 | 0.766784452 | 0.233215548 |

* The data of sex and age-specific death rate between ages x and x+n come from the China Life Table of 2013 at the WHO website [1].

† The data of age-specific sex structure come from the China Population and Employment Statistics Yearbook 2014 [2].

# **2. The NCMS’s impact on mortality**

These assumptions were tested using the average effects of the program throughout rural China. We used the estimate of the NCMS’s impact on mortality from Zhou and his colleagues’ paper [3]. The coefficients listed in Table B are the estimated marginal effects of NCMS on age-adjusted death rates by gender and age group per 1000 population. We then took these numbers (multiplied by 1000) and, via the model, added them to the mortality rate for the corresponding gender and age group to represent the effect of NCMS.

**Table B. Estimated marginal effect of the NCMS by age group and gender per 1000 population**

| **Age, gender** | **Age 20-44** | | **Age 45-59** | | **Age 60+** | |
| --- | --- | --- | --- | --- | --- | --- |
|  | **Male** | **Female** | **Male** | **Female** | **Male** | **Female** |
| Cof. | -0.10 | -0.05 | -0.14 | -0.05 | 0.36 | 1.05 |
| S.E. | 0.09 | 0.04 | 0.22 | 0.14 | 1.72 | 1.41 |
| 95% CI Min | -0.28 | -0.12 | -0.57 | -0.32 | -3.01 | -1.72 |
| 95% CI Max | 0.08 | 0.03 | 0.29 | 0.23 | 3.73 | 3.82 |

# **3. The incidence of being hypertensive**

The sex-specific prevalence of hypertension in the age group of 20 to 30 years was about 9.7% in males and 3.8% in females in China [4]. Therefore, we assumed that the 20-year-old male and female in the hypothetical cohort had a 9.7% and 3.8% respectively of being hypertensive. Liang et al (2014) estimated the age-specific incidence of hypertension among Chinese aged at least 18 years between 2004 and 2009 (See Table C) [5]. We assumed that each person contributes 1 person-year to the denominator of the incidence and the hypothetical cohort was exposed to the age-specific probability of hypertension after 20 years old.

**Table C. Age-specific incidence of hypertension among Chinese adults (age>=18 years) between 2004 and 2009 (n=4523)**

| **Age, years** | **Incidence rate of hypertension, per 100 person-years** |
| --- | --- |
| 18-39 | 2.6 |
| 40-59 | 5.7 |
| 60 or above | 11.0 |
| Source: Adjusted from Liang et al., 2014, International Journal of Cardiology. | |

# **4. The estimation of total health expenditure per rural resident by provincial region**

The NCMS is administered at the county level. Each country is responsible for designing benefit packages and implementing their own health plans. Therefore, the total health expenditure per rural resident (including out-of-pocket, social, and governmental expenditures) might vary by county. However, we could not get county-level data. In our analyses, we considered the provincial difference of total health expenditure per rural resident. According to the 2014 Health Statistical Yearbook, the total health expenditure per rural resident and per urban resident at the national level in 2013 is 1274 CNY and 3234 CNY, respectively [6]. The yearbook also reported the total health expenditure per person by provincial region in 2013 (See the second column in Table D) [6]. Since it did not report the total health expenditure by urban/rural residents for each provincial region, we estimated the total health expenditures per rural resident by provincial area based on the ratio of total health expenditure per urban resident to total health expenditure per rural resident, the ratio of urban population to rural population, and the total health expenditure per person by provincial region. We reported estimated total health expenditure per rural resident by provincial region in the third and fourth column of S4 Table. Beijing had the highest total health expenditure per rural resident ($977 in PPP; $559 in nominal), while Guizhou had the lowest estimate ($243 in PPP; $139 in nominal).

**Table D. The total health expenditure per rural resident by provincial region in 2013**

| **Regions** | **Total health expenditure per person** ^a^ **in constant 2013 US dollars after the purchasing power parities (PPP) adjustment** * | **Estimated total health expenditure per rural resident in constant 2013 US dollars after the PPP adjustment** † | **Estimated total health expenditure per rural resident in constant 2013 US dollars based on average exchange rate (nominal conversion)** ‡ |
| --- | --- | --- | --- |
| Nation | 656 | 359 | 205 |
| Beijing | 1785 | 977 | 559 |
| Tianjin | 1056 | 578 | 331 |
| Hebei | 571 | 312 | 179 |
| Shanxi | 569 | 311 | 178 |
| Inner Mongolia | 788 | 431 | 247 |
| Liaoning | 755 | 413 | 237 |
| Jilin | 783 | 428 | 245 |
| Heilongjiang | 711 | 389 | 223 |
| Shanghai | 1456 | 797 | 456 |
| Jiangsu | 785 | 430 | 246 |
| Zhejiang | 877 | 480 | 275 |
| Anhui | 571 | 312 | 179 |
| Fujian | 623 | 341 | 195 |
| Jiangxi | 460 | 252 | 144 |
| Shandong | 650 | 356 | 204 |
| Henan | 505 | 276 | 158 |
| Hubei | 598 | 327 | 187 |
| Hunan | 550 | 301 | 172 |
| Guangdong | 667 | 365 | 209 |
| Guangxi | 506 | 277 | 158 |
| Hainan | 582 | 319 | 182 |
| Chongqing | 699 | 383 | 219 |
| Sichuan | 582 | 319 | 182 |
| Guizhou | 444 | 243 | 139 |
| Yunnan | 509 | 279 | 160 |
| Xizang | 666 | 365 | 209 |
| Shaanxi | 761 | 416 | 238 |
| Gansu | 565 | 309 | 177 |
| Qinghai | 792 | 434 | 248 |
| Ningxia | 724 | 396 | 227 |
| Xinjiang | 830 | 454 | 260 |

* The total health expenditure per person by provincial region in 2013 were obtained from the 2014 Health Statistical Yearbook [6].

† The purchasing power parities exchange rate in 2013 (1 USD≈3.55 CNY) was obtained from the World Bank [7].

‡ The average exchange rate in 2013 (1 USD≈6.20 CNY) was obtained from World Bank [8].

# **5. The NCMS’s effect on labor force participation**

Shen et al (2017) found that the NCMS increased the number of hours worked in agriculture and the probability of off-farm labor participation among rural residents aged at least 30 years old (See Table E) [9]. The number of hours worked in agriculture (556 hours per year) and the probability of off-farm labor participation (16.5%) among the non-NCMS group during the post period of 2006 were considered as the baseline values in the control group [9].

**Table E. The effects of NCMS on labor force participation by age groups in rural China**

|  | **Increase in the number of hours worked in agriculture due to the NCMS** | **Increase in the likelihood of off-farm labor participation due to the NCMS** |
| --- | --- | --- |
| Age between 30-49 years | 1.097 | 0.134 |
| Age 50 years or more | 1.280 | 0.069 |
| Source: Shen et al., 2017, China Agricultural Economic Review. | | |

According to the 2015 China Rural Statistical Yearbook [10], the annual income in agriculture per rural resident was about $800 in 2013 after the purchasing power parity (PPP) adjustment ($458 in nominal). Jilin had the highest annual income in agriculture ($1,442 in PPP; $825 in nominal), while Shanghai had the lowest estimate ($260 in PPP; $149 in nominal). To calculate the hourly income in agriculture, the annual income in agriculture per rural resident was divided by the average number of hours worked in agriculture among the control group (556 hours per year). To estimate the additional income from the agricultural production due to the NCMS, we multiplied the increase in hours worked in agriculture by the hourly income in agriculture.

According to the 2015 China Rural Statistical Yearbook [10], the annual wage per rural resident was about $1,029 in 2013 after the PPP adjustment ($589 in nominal). The annual wage per rural resident was the highest in Shanghai ($4,198 in PPP; $2,404 in nominal) and lowest in Xinjiang ($350 in PPP; $200 in nominal).

In the model, we entered the following variables: the increase in hours worked in agriculture, the hourly income in agriculture, the probability of off-farm labor participation in the control group, the increase in the likelihood of off-farm participation due to the NCMS, and the annual wage per rural resident. We reported the baseline, the low and high values in Table 1 of the manuscript.

# **6. Supporting information on the cost-effectiveness analysis**

## 6.1 Results of probabilistic sensitivity analysis

In the probabilistic sensitivity analysis with Monte Carlo simulations (Table F), the gains for the average participant were approximately 0.01 (95% CI: -0.08, 0.10) QALYs, and the additional cost attributable to the NCMS was Int$1,013 per rural resident (95% CI: -2,773, 5,363), indicating that the ICER was Int$66,698 (95% CI: cost-saving, Int$845,658) per QALY gained.

**Table F. Results of probabilistic sensitivity analysis with** **Monte Carlo simulations on the NCMS versus no NCMS (NCMS = New Rural Medical Cooperative System)**

|  | **Mean** | **95% CI *** |
| --- | --- | --- |
| Incremental cost, Int$ † | 1013 | (-2773, 5363) |
| Incremental effectiveness, QALYs ‡ | 0.01 | (-0.08, 0.1) |
| ICER, Int$ †/QALYs ‡ | 66,698 | (Cost-saving, 845,658) |

* CI = confidence interval.

† Int$ = international dollars. 1 International dollar=3.55 CNY.

‡ QALY = quality-adjusted life years.

## 6.2 Incremental cost-effectiveness scatterplot report

Table G presents results generated from a Monte Carlo simulation (10,000 simulations of probabilistic sensitivity analysis). According to the results, the ICER of implementing the NCMS was more likely to exceed the willingness-to-pay (WTP) threshold—three times per capita GDP of China in 2013 ($37,059). The probabilities of the NCMS being dominant and cost-effective were 17.5% and 15.3%, respectively. The ICER of implementing the NCMS had a 34.8% chance of exceeding the WTP threshold and a 23.5% chance of reducing quality-adjusted life years and incurring costs.

**Table G. The incremental cost-effectiveness scatterplot report***

| **Component** | **Quadrant** | **INCREFF** | **INCRCOST** | **INCRCE** | **Frequency** | **Proportion** |
| --- | --- | --- | --- | --- | --- | --- |
| C1 | IV | IE>0 | IC<0 | Superior | 1751 | 0.1751 |
| C2 | I | IE>0 | IC>0 | ICER<$37,059 | 1527 | 0.1527 |
| C3 | III | IE<0 | IC<0 | ICER>$37,059 | 509 | 0.0509 |
| C4 | I | IE>0 | IC>0 | ICER>$37,059 | 3480 | 0.3480 |
| C5 | III | IE<0 | IC<0 | ICER<$37,059 | 384 | 0.0384 |
| C6 | II | IE<0 | IC>0 | Inferior | 2349 | 0.2349 |
| Indiff | origin | IE=0 | IC=0 | 0/0 | 0 | 0 |

* Three times per capita GDP of China in 2013 was used as the willingness-to-pay threshold.

## 6.3 One-way sensitivity analyses

Table H presents results obtained from a series of one-way sensitivity analyses. According to the results, the NCMS remained not cost-effective across the range of health-related quality of life score among normotensives and hypertensives, hypertension-related medical costs per year, additional hours worked in agriculture, and hourly income in agriculture.

**Table H. One-way sensitivity analyses, NCMS versus no NCMS (NCMS=New Rural Cooperative Medical Scheme)**

| **Parameters** | **Incremental cost** | | **Incremental effectiveness** | | **Incremental cost-effectiveness ratio** | |
| --- | --- | --- | --- | --- | --- | --- |
|  | **Low** | **High** | **Low** | **High** | **Low** | **High** |
| Health-related quality of life score among normotensives | 909 | 909 | 0.01 | 0.01 | 77,146 | 72,957 |
| Health-related quality of life score among hypertensives | 909 | 909 | 0.01 | 0.01 | 65,734 | 87,289 |
| Annual direct medical costs of hypertension among rural residents | 914 | 904 | 0.01 | 0.01 | 75,419 | 74,565 |
| Increase in hours of working in agriculture per year, hour |  |  |  |  |  |  |
| Aged 30-49 years | 912 | 906 | 0.01 | 0.01 | 75,244 | 74,719 |
| Aged 50 years and above | 911 | 907 | 0.01 | 0.01 | 75,133 | 74,845 |
| Hourly income in agriculture | 924 | 893 | 0.01 | 0.01 | 76,225 | 73,690 |

# **7. Reference**

1. World Health Organization (WHO). Life tables by country China. 2015. Available from: <http://apps.who.int/gho/data/view.main.60340?lang=en>.

2. China National Bureau of Statistics (CNBS). China Population and Employment Statistics Yearbook Beijing: China Statistics Press, 2014.

3. Zhou M, Liu S, Bundorf K, Effleston K, Zhou S. Mortality in Rural China Declined as Health Insurance Coverage Increased, But No Evidence the Two are Linked. Health Affairs. 2017; 36(9): 1672-1678.

4. Gao Y, Chen G, Tian H, Lin L, Lu J, Weng J, et al. Prevalence of hypertension in China: a cross-sectional study. PLoS One. 2013;8(6): e65938.

5. Liang Y, Liu R, Du S, Qiu C. Trends in incidence of hypertension in Chinese adults, 1991–2009: the China Health and Nutrition Survey. Int J Cardiol. 2014;175(1):96-101.

6. Minister of Health of the People’s Republic of China (CMOH). China Health Statistical Yearbook. Beijing: Peking Union Medical College; 2014.

7. The World Bank Group. PPP conversion factor, GDP (LCU per international $). <https://data.worldbank.org/indicator/PA.NUS.PPP> (Accessed on 23 April 2018).

8. The World Bank Group. Official exchange rate (LCU per US$, period average). <https://data.worldbank.org/indicator/PA.NUS.FCRF> (Accessed on 23 April 2018).

9. Shen Z, Parker M, Brown D, Fang X. Effects of public health insurance on labor supply in rural China. China Agricultural Economic Review. 2017;9(4):623-42.

10. China National Bureau of Statistics (CNBS). 2015 China Rural Statistical Yearbook. Beijing: China Statistics Press; 2016.
